# Isolated left ventricular non-compaction as an unusual cause of heart failure: a case report

**DOI:** 10.1186/1752-1947-2-269

**Published:** 2008-08-13

**Authors:** Nicholas LM Cruden, Martin A Denvir

**Affiliations:** 1 Department of Cardiology, Royal Infirmary of Edinburgh, Little France Crescent, Little France, Edinburgh, EH16 5SA, UK

## Abstract

**Introduction:**

Isolated left ventricular non-compaction is a recently described form of cardiomyopathy that is associated with a significant risk of life-threatening arrhythmia and thromboembolic complications.

**Case presentation:**

We report the presentation, diagnosis and management of isolated left ventricular non-compaction in a 54-year-old Caucasian woman presenting with progressive symptoms of heart failure.

**Conclusion:**

Advances in diagnostic imaging have undoubtedly led to an increase in the detection of isolated left ventricular non-compaction. Diagnosing and differentiating this uncommon condition from other forms of cardiomyopathy are important as treatment and prognosis may differ significantly. Our current understanding of isolated left ventricular non-compaction, including diagnostic criteria, management and prognosis, is discussed.

## Introduction

During normal foetal development, myocardial compaction usually occurs by day 70 *in utero*. Where this process fails to take place, prominent left ventricular trabeculations may remain that persist into adult life. In the absence of significant cardiac outflow tract obstruction, the presence of extensive left ventricular trabeculation is associated with the development of left ventricular systolic impairment, cardiac arrhythmias and systemic thromboembolism. Recent advances in diagnostic imaging techniques have led to an increase in the detection of this previously rare form of cardiomyopathy, known as isolated left ventricular non-compaction (IVNC). It is important that clinicians recognise and differentiate this condition from other forms of cardiomyopathy as treatment and prognosis may differ significantly.

## Case presentation

A 54 old-year-old Caucasian woman was admitted with a 3-month history of progressive exertional breathlessness, orthopnoea and chest tightness. On examination she was in sinus rhythm with a rate of 66 beats/minute and a blood pressure of 90/60 mmHg. Auscultation revealed a first and second heart sound with no added sounds and no murmurs, reduced air entry at both lung bases and coarse crepitations at the left lung base.

**Figure 1:**
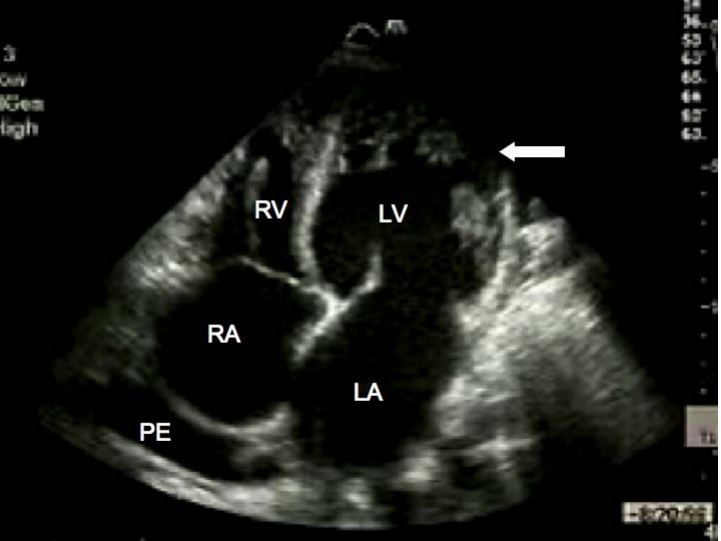
**Transthoracic echocardiography**. Apical four chamber view demonstrating marked trabeculation of the left ventricular apex (arrow). RA, right atrium; LA, left atrium; LV, left ventricle; RV, right ventricle; PE, pleural effusion.

**Figure 2:**
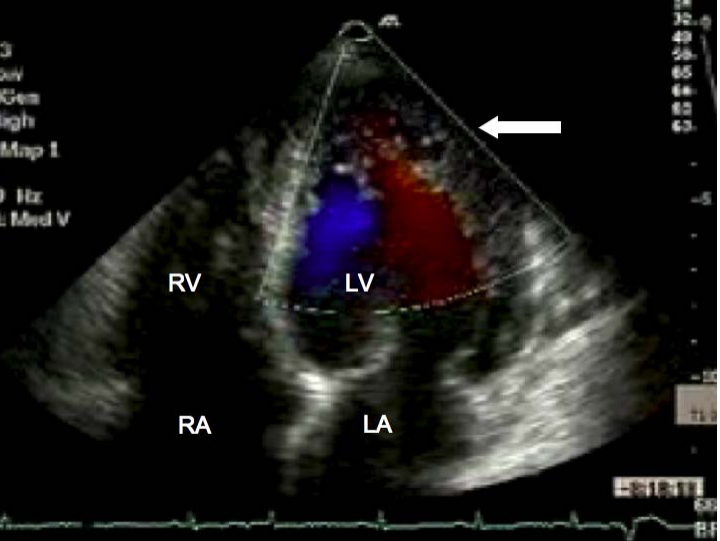
**Transthoracic echocardiography.** Doppler colour flow mapping suggesting blood flow present between the ventricular trabeculations (arrow). RA, right atrium; LA, left atrium; LV, left ventricle; RV, right ventricle.

**Figure 3:**
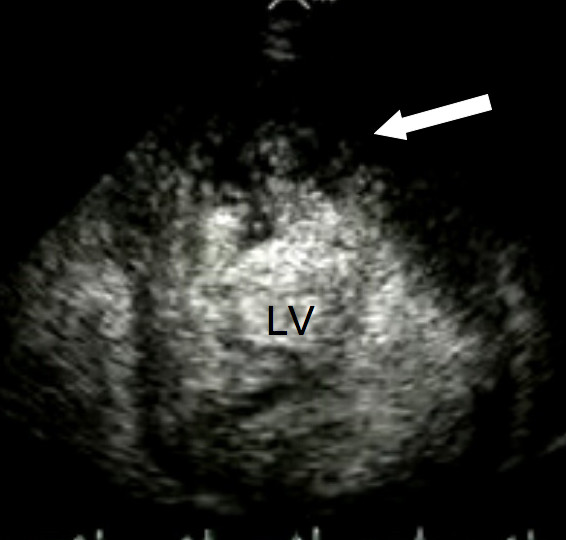
**Transthoracic echocardiography**. Following intravenous injection, contrast agent is visualised between the ventricular trabeculations (arrow). LV, left ventricle.

Serum urea, electrolytes, thyroid function, ferritin and full blood count were all within normal limits. A chest X-ray demonstrated cardiomegaly with small bilateral pleural effusions. The electrocardiogram confirmed sinus rhythm with left atrial enlargement, low voltage QRS complexes and lateral T wave inversion. Transthoracic echocardiography demonstrated a dilated left ventricle (end systolic diameter 5.5 cm; end diastolic diameter 5.9 cm) with severe systolic impairment and hypertrabeculation of the left ventricular apex (Fig. 1) in the absence of significant valvular heart disease. Doppler colour flow mapping confirmed colour flow between the trabeculations (Fig. 2). Intravenous injection of ultrasound contrast agent confirmed an area of non-compacted myocardium subtending a thinner walled area of compaction and a diagnosis of IVNC was made (Fig. 3).

## Discussion

Isolated left ventricular non-compaction is a recently described cardiomyopathy [1], the true prevalence of which remains unknown. Advances in diagnostic imaging modalities have undoubtedly led to an increase in detection of this rare condition and it is likely that earlier cases have been misdiagnosed as phenotypically similar cardiomyopathies, such as apical hypertrophic cardiomyopathy [2], where prognosis and treatment may differ significantly. The purpose of this case report is to highlight the diagnosis of IVNC and briefly review our current understanding of the condition.

The presence of marked left ventricular trabeculation in patients with IVNC is believed to arise as a result of intrauterine arrest of left ventricular myocardial compaction, although the trigger for this phenomenon is not yet known. Both familial and sporadic forms of IVNC have been described and although no causative gene has yet been identified, familial screening is recommended [3,4].

Echocardiography remains the reference standard for the diagnosis of IVNC [5]. Jenni and colleagues identified four criteria for the diagnosis of IVNC by echocardiography [5]. A thick, inner layer of non-compacted myocardium is present subtending an outer, thin compacted layer of myocardium with ratio of non-compacted to compacted myocardium during systole being greater than 2:1. When the left ventricle is divided into nine segments, non-compacted myocardium is present predominantly (more than 80%) on the apical and mid-ventricular aspects of the inferior and lateral walls. Deeply perfused intertrabecular recesses that do not communicate with the coronary circulation can be identified using colour Doppler (Fig. 2). The use of an intravenous ultrasound contrast agent (Fig. 3) or Doppler tissue imaging may improve visualisation of the left ventricular intertrabecular recesses. These features should be present in the absence of any other significant cardiac abnormality.

Cardiac magnetic resonance imaging may also be of use in the diagnosis of IVNC, in particular in individuals where the image quality at echocardiography is limited [6]. In contrast to echocardiography, with cardiac magnetic resonance imaging the ratio of non-compacted to compacted myocardium should be measured during diastole, a ratio of greater than 2.3:1 confirming pathological trabeculation of the left ventricle [6].

Treatment for patients with IVNC should be directed at the management of left ventricular systolic impairment where present; the detection, treatment and prevention of arrhythmias; and the prevention of systemic embolic events [3,4]. In addition to treatment with angiotensin-converting enzyme inhibitors, β-blockers and, where appropriate, diuretics and/or digoxin, all patients with IVNC should be screened annually with 24-hour electrocardiogram recordings and considered for long-term prophylactic anticoagulation with warfarin. The high incidence of sudden death reported in patients with IVNC has prompted some authors to advocate a strategy of "early" automated implantable cardiodefibrillator implantation [3]. The role of biventricular pacemakers in this population remains unclear. Finally, where pharmacological therapy fails to halt the progression to cardiac failure, heart transplantation should be considered [4].

Initial data from Europe and America reported a 4- to 6-year combined mortality or transplantation rate of ~50% to 60% [3,4] although recent UK data indicate the prognosis may be more favourable [7]. Our patient responded well to the introduction of angiotensin-converting enzyme inhibition, beta-blockade and warfarin anticoagulation but is currently being considered for cardiac transplantation.

## Conclusion

Isolated left ventricular non-compaction is a rare but important form of cardiomyopathy that should not be overlooked in patients presenting with cardiac failure. This case report emphasises the importance of differentiating this condition from alternative diagnoses where treatment and prognosis may vary significantly.

## Abbreviations

IVNC: Isolated left ventricular non-compaction.

## Competing interests

The authors declare that they have no competing interests.

## Authors' contributions

NLMC and MAD made the diagnosis and were involved in subsequent management. MAD was the Consultant responsible for the patient. Both authors have drafted, read and approved the manuscript.

## Consent

Written informed consent was obtained from the patient for publication of this case report and accompanying images. A copy of the written consent is available for review by the Editor-in-Chief of this journal.
